# Supplementary material for: Impact of Obesity on Clinical Outcomes of Patients with Intra-Abdominal Hypertension and Abdominal Compartment Syndrome
Source: Life (Basel). 2023 Jan 24;13(2):330. doi: 10.3390/life13020330 (PMC9961081; doi:10.3390/life13020330)
Supplement: Supplementary file 1 [file life-13-00330-s001.zip › life-2155151-supplementary.pdf]

Supplementary Materials

Supplementary Table S1: Search strategies used in various databases

Database	Search strategy
PubMed	((Intra-abdominal hypertension[MeSH Terms]) OR (“Intra Abdominal Hypertension”[Title/Abstract]) OR (“Intra Abdominal Hypertension”[Other Term]) OR (“Intraabdominal Hypertension” [Title/Abstract]) OR (“Intraabdominal Hypertension” [Other Term]) OR (“Intra-Abdominal Pressure” [Title/Abstract]) OR (“Intra-Abdominal Pressure” [Other Term]) OR (“Intraabdominal Pressure” [Title/Abstract]) OR (“Intraabdominal Pressure”[Other Term]) OR (“Abdominal Pressure” [Title/Abstract]) OR (“Abdominal Pressure” [Other Term]) OR (“Abdominal Hypertension” [Title/Abstract]) OR (“Abdominal Hypertension” [Other Term]) OR (“Abdominal Compartment Syndrome” [Title/Abstract]) OR (“Abdominal Compartment Syndrome” [Other Term])) AND ((Obesity[MeSH Terms]) OR (Obesity, Abdominal[MeSH Terms]) OR (Obesity, Morbid[MeSH Terms]) OR (Obese[Title/Abstract]) OR (Obese[Other Term]) OR (Overweight[Title/Abstract]) OR (Fat[Title/Abstract]) OR (Fat[Other Term]) OR (“body mass”[Title/Abstract]) OR (“body mass”[Other Term]) OR (“body mass index”[Title/Abstract]) OR (“body mass index”[Other Term]) OR (BMI[Title/Abstract]) OR (BMI[Other Term]))
Embase (Ovid)	("intra-abdominal hypertension" or "intra abdominal hypertension" or "intraabdominal hypertension" or "abdominal pressure" or "abdominal hypertension" or "abdominal compartment syndrome").ti,ab,kw. AND (obesity or "abdominal obesity" or "morbid obesity" or overweight or fat or "body mass" or "body mass index" or "bmi").ti,ab,kw.
Scopus	TITLE-ABS-KEY ("intra-abdominal hypertension" OR "intra abdominal hypertension" OR "intraabdominal hypertension" OR "abdominal pressure" OR "abdominal hypertension" OR "abdominal compartment syndrome") AND TITLE-ABS-KEY (obesity OR "abdominal obesity" OR "morbid obesity" OR overweight OR fat OR "body mass" OR "body mass index" OR "bmi")
